# Curare

**Published:** 1916-09

**Authors:** 


### CURARE

The reputed action of curare to produce motor paralysis while sensation remains unimpaired, being out of analogy to the action of most drugs and supported mainly by tradition and fine writing, has seemed to us worth investigation. Curare is a strong "talking point" with antivivisectionists, although it is seldom used at present and obviously should not be if its action is as reputed, unless very urgent reasons counterbalance the demand for mercy. Support of the peculiar action claimed for curare can obviously depend only on the statement of human beings who have recovered from the poison, which is usually fatal in large dose and very little employed therapeutically in small dose. In small dosage, any narcotic may fail of its purpose to deaden pain perfectly and allowance must be made for this fact and the general imperfections of subjective evidence. Out of a considerable number of inquiries made, only the two following replies have been received, except that some have written that they were unable to speak from positive knowledge and have not employed curare in animal experimentation.

**University of London**
King's College
Strand, W. C., 7-28-'16.

The cases in man are so rare, that it is not safe to assume that curare kills sensation. There are cases which show it has such an effect, but it is safest not to regard it as an anesthetic. It is not so regarded by the English law, and in those rare cases where its use is necessary, it can always be combined with the administration of some anesthetic concerning the power of which there is no doubt.

W. D. HALIBURTON.

---

**Harvard Medical School**
240 Longwood Avenue, Boston, Mass.

July 24, 1916.

Dear Dr. Benedict:

There is in the literature a conflict of evidence regarding the action of curare on afferent nerves. The earlier observations led to the conclusion that it might be a sensitizer, at least of the spinal cord, so that it would have an effect something like strychnine so far as afferent nerves are concerned. Later evidence has indicated that it is, in sufficiently large doses, depressant for all active structures. I have written to a number of the most eminent pharmacologists and they have all testified that, under the circumstances, they would not trust to its anesthetic action, but would invariably employ along with it some anesthetic drug.

You are right in understanding that curare is relatively rarely used by experimenters. So far as I know, the testimony of the pharmacologists, which I have given above, expresses the spirit in which workers generally would employ curare. I have recently employed it myself in a few experiments, but only after decerebration under ether.

Yours sincerely,

W. B. CANNON.

